# Transcatheter aortic valve replacement: Past, present, and future

**DOI:** 10.1002/clc.24209

**Published:** 2024-01-14

**Authors:** Akash Srinivasan, Felyx Wong, Brian Wang

**Affiliations:** ^1^ Division of Medical Sciences, Nuffield Department of Surgical Sciences University of Oxford Oxford UK; ^2^ Guy's and St Thomas’ NHS Foundation Trust London UK; ^3^ Department of Metabolism, Digestion and Reproduction, Faculty of Medicine Imperial College London London UK

**Keywords:** aortic stenosis, prostheses, surgery, transcatheter aortic valve replacement (TAVR), valvular heart disease

## Abstract

Transcatheter aortic valve replacement (TAVR) has emerged as a ground‐breaking, minimally invasive alternative to traditional open‐heart surgery, primarily designed for elderly patients initially considered unsuitable for surgical intervention due to severe aortic stenosis. As a result of successful large‐scale trials, TAVR is now being routinely applied to a broader spectrum of patients. In deciding between TAVR and surgical aortic valve replacement, clinicians evaluate various factors, including patient suitability and anatomy through preprocedural imaging, which guides prosthetic valve sizing and access site selection. Patient surgical risk is a pivotal consideration, with a multidisciplinary team making the ultimate decision in the patient's best interest. Periprocedural imaging aids real‐time visualization but is influenced by anaesthesia choices. A comprehensive postprocedural assessment is critical due to potential TAVR‐related complications. Numerous trials have demonstrated that TAVR matches or surpasses surgery for patients with diverse surgical risk profiles, ranging from extreme to low risk. However, long‐term follow‐up data, particularly in low‐risk cases, remains limited, and the applicability of published results to younger patients is uncertain. This review delves into key TAVR studies, pinpointing areas for potential improvement while delving into the future of this innovative procedure. Furthermore, it explores the expanding role of TAVR technology in addressing other heart valve replacement procedures.

## Graphical abstract

In this review, we provide an updated overview of the TAVR procedure, the main aspects of preprocedural patient assessment, and the key complications of the procedure. We also summarize the current evidence comparing TAVR and SAVR, before exploring the potential future directions for TAVR. Created with BioRender.

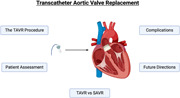

**Abbreviations**
ACC/AHA: American College of Cardiology/American Heart Association
CEP: cerebral embolic protection
CVE: cerebrovascular event
ER: extreme risk
ESC/EACTS: European Society of Cardiology/European Association for Cardio‐Thoracic Surgery
EuroSCORE II: European System for Cardiac Operative Risk Evaluation II
HR: high risk
LR: low risk
MDCT: multidetector computed tomography
NOTION: nordic aortic valve intervention
PARTNER: placement of aortic transcatheter valve
PPM: permanent pacemaker
SFAR: sheath‐to‐femoral‐artery ratio
SMART: SMall Annuli Randomized To Evolut or SAPIEN
SOLVE‐TAVI: compariSon of secOnd‐generation seLf‐expandable versus balloon‐expandable Valves and gEneral versus local anaesthesia in Transcatheter Aortic Valve Implantation
STS‐PROM: Society of Thoracic Surgeons Predicted Risk of Mortality
SURTAVI: surgical replacement and transcatheter aortic valve implantation
TAVR: transcatheter aortic valve replacement
TMVR: transcatheter mitral valve replacement
TOE: transoesophageal echocardiography
TTE: transthoracic echocardiography

## 1 BACKGROUND

Aortic stenosis is present in over 2% of adults over the age of 65, making it the most common valvular heart disease in the developed world.^1^ A further 25% of the population aged 65 or more have aortic sclerosis.^2^ For this reason, together with the ageing population, the impact of aortic valve disease on healthcare resources is expected to increase. Aortic sclerosis is associated with a 50% increase in cardiovascular mortality^1^ and hence optimizing the management of aortic valve disease is considered a priority for the cardiovascular field.

The earliest experimental approaches towards treating valvular disease include the digital dilation of a stenosed aortic valve, performed by Theodore Tuffier in 1912. Subsequently, open‐heart surgery was the only option available to patients with significant valvular disorders for the best part of a century.^3^ Procedures evolved tremendously after the introduction of cardiopulmonary bypass,^4^ but surgical valve replacement has always carried a risk of mortality. This risk is higher in patients with comorbidities such as renal insufficiency or vascular disease, of which many have calcific aortic stenosis.^5^ As a result, the 2003 Euro Heart Survey found that over 30% of patients with a severe valvular disease did not receive intervention, primarily due to comorbidities.^5^

A significant breakthrough was achieved in 2002 when Alain Cribier performed the first transcatheter aortic valve replacement (TAVR) by taking an antegrade approach from the right femoral vein.^6^ This initial procedure resulted in severe noncardiac complications such as pulmonary embolism, lower limb ischaemia and subsequent death,^6^ but it has rapidly grown in popularity over the last two decades.^7^, ^8^ In 2019, TAVR was performed more frequently in the United States than surgical aortic valve replacement (SAVR).^9^

In recent years, the clinical uses of TAVR have been expanded due to advances in valve‐in‐valve TAVR technology, serving as a feasible alternative to redo surgery.^10^ Patients necessitating another valve procedure are often associated with higher surgical risks and may be unsuitable for further surgery due to adhesions,^11^ which highlights the importance of alternatives such as the valve‐in‐valve TAVR. Today, TAVR has become an established procedure which serves as a viable alternative when patients are unsuitable for surgical replacement,^12^ and it is sometimes preferred for patients with a lower surgical risk due to its less invasive nature.^13^, ^14^

We provide an overview of the TAVR procedure, the pivotal role of imaging in its peri‐procedural management, and the key complications associated with the intervention. We also compare TAVR with the conventional surgical procedure and explore what the future holds for TAVR and transcatheter approaches for treating diseases involving other valves.

## 2 THE TAVR PROCEDURE

Since the first TAVR in 2002, the procedure has evolved significantly in its technique, the access points, and the choice of anaesthesia. Cribier performed the first TAVR on a 57‐year‐old male with significant co‐morbidities who was placed under mild, conscious sedation and local anaesthesia.^6^ After this first attempt, further measures were introduced including general anaesthesia and intraprocedural transoesophageal echocardiography (TOE). The drawbacks include a requirement for endotracheal intubation and ventilation, haemodynamic instability and longer hospital stays.^15^ Consequently, the “minimalist” combination of local anaesthesia and conscious sedation demonstrated by Cribier has since been revisited,^16^ and there has been an increased focus on simplifying the TAVR procedure.

### 2.1 Anaesthetic approaches

The anaesthetic approaches to TAVR have been compared mostly through nonrandomized trials and registry data. In 2008, a case series by Behan et al.^17^ suggested the potential benefits of TAVR with sedation, which include a shorter stay in high‐dependency areas and fewer complications. Conversely, in the same year, Ree et al.^18^ reported adverse experiences when using sedation alone after four patients needed unplanned vascular surgery to repair the TAVR access site, which necessitated conversion to general anaesthesia. More recent studies have suggested that local anaesthesia with conscious sedation is a feasible approach with a 2018 registry analysis by Eskandari et al.^19^ concluding that procedural outcome, 30‐day and 1‐year mortality are not affected by the anaesthetic approach. It has become apparent that general anaesthesia is associated with a longer procedure duration and hospital stay.^19^ From a managerial perspective, this may also have cost implications on patient care.^20^ Several centers have therefore adopted a minimalist approach for patients necessitating TAVR.^21^

### 2.2 Access sites

The femoral access remains the preferred approach.^22^ and technological improvements have reduced the sheath sizes from 24–25 Fr down to 14–16 Fr,^23^ thus decreasing bleeding complications.^24^ The transfemoral approach is the least invasive since it is usually performed completely percutaneously, and therefore it is the most permissive to local anaesthesia and sedation.^25^ However, alternative nonfemoral access sites are sometimes used, as seen in 28.8% of cases reported by the UK TAVI registry.^25^ These routes are typically chosen when femoral access is limited by factors such as tortuous iliofemoral vasculature and obstructive peripheral vascular disease.^26^

**Figure 1:**
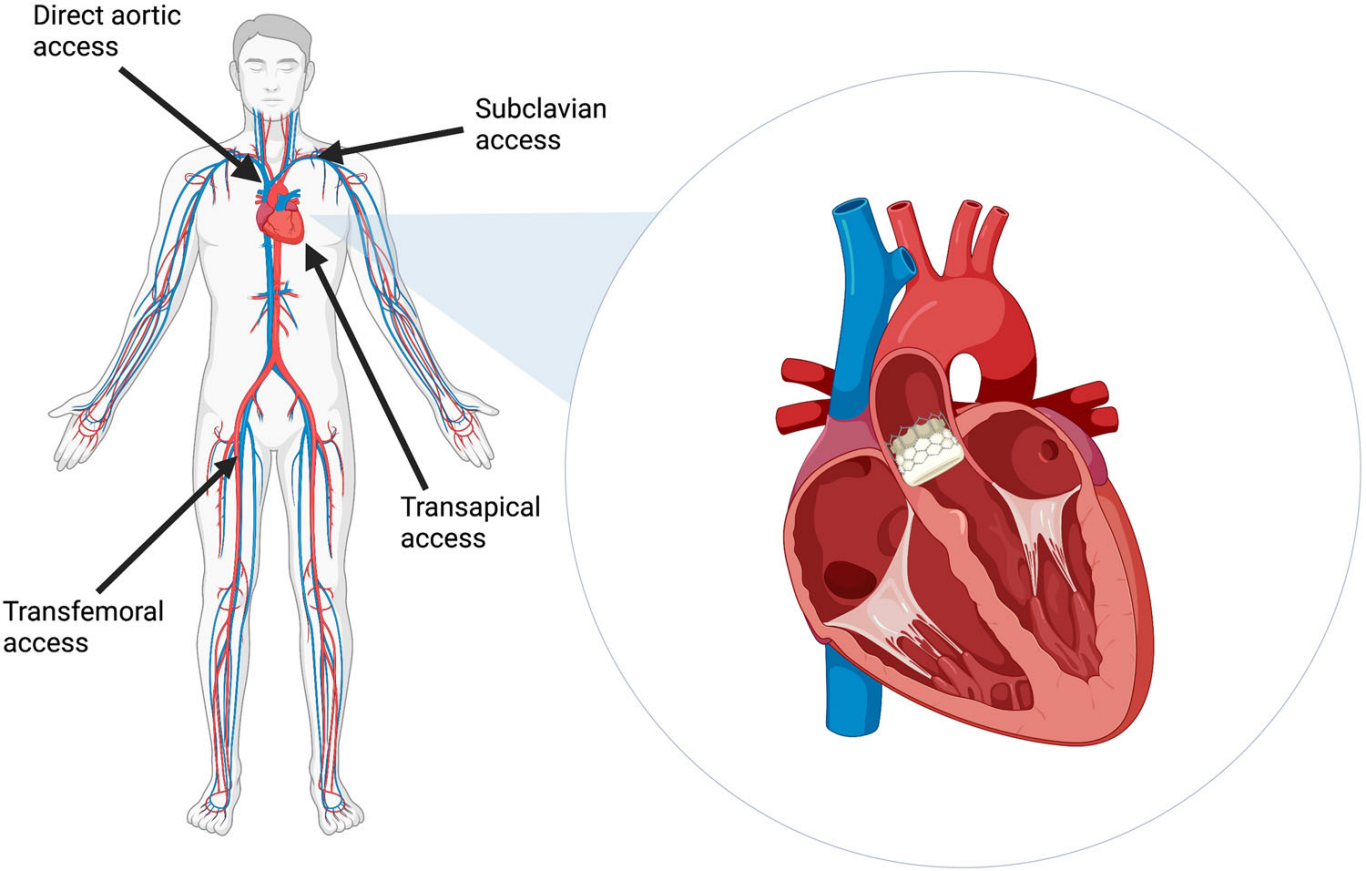
Access points for the TAVR procedure. Created with BioRender. TAVR, transcatheter aortic valve replacement.

One of the most established nonfemoral alternatives is the transapical route,^25^, ^27^ which involves performing a left mini‐thoracotomy before puncturing the apex to deliver the valve system.^26^ This approach has been associated with fewer vascular complications than the transfemoral route, but a higher rate of all‐cause mortality.^28^ Less common methods include the direct aortic access.^29^ and the transaxillary/subclavian approaches,^30^ which were both used in approximately 5% of cases in the UK TAVI registry.^25^ These methods also require surgery to expose and access the artery, although a percutaneous TAVR using the trans‐axillary route has been described in the literature with no major complications relating to the access site.^31^ Figure 1 depicts a visual representation of the various access routes available for TAVR.

### 2.3 Valve systems

There are multiple heart valve systems used for the TAVR procedure. First, Medtronic has developed a series of Evolut heart valves, which are composed of porcine tissue attached to a self‐expanding nickel titanium frame.^32^ A delivery catheter is used to insert and release these artificial valves into the body, before they self‐expand and attach to the damaged heart valve.^32^ The Sapien 3 and Sapien 3 Ultra valves designed by Edwards Lifesciences are made of bovine tissue and attached to a cobalt‐chromium frame.^33^ However, these valves are delivered using a balloon catheter and expanded by the balloon, before anchoring to the damaged aortic valve.^33^

**Figure 2:**
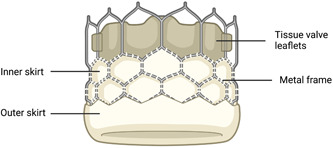
Simplified schematic depiction of the outer skirt as seen in the more recent generation transcatheter aortic valve replacement implants. Created with BioRender.

Heart valve systems have evolved significantly over the past decade. First‐generation devices, such as the Edwards Sapien and the Medtronic CoreValve, demonstrated efficacy in early trials. Unfortunately, they were also linked with issues such as paravalvular aortic regurgitation, vascular complications, conduction disturbances and stroke, thus leading to poorer prognoses.^34^, ^35^, ^36^ However, technological advances have focused on reducing such complications; for example, smaller delivery sheaths are used to reduce the degree of vascular trauma and bleeding events, whilst the addition of outer skirts has helped to prevent paravalvular regurgitation (Figure 2).^34^

Currently, the literature supports the use of newer generation versions of both self‐expandable.^37^ and balloon‐expandable.^38^ valves, but there is limited data comparing these two options. A small trial of 241 patients receiving TAVR with first‐generation valve systems found that balloon‐expandable devices had a higher success rate.^39^ Nevertheless, the more recent compariSon of secOnd‐generation seLf‐expandable versus balloon‐expandable Valves and gEneral versus local anaesthesia in Transcatheter Aortic Valve Implantation (SOLVE‐TAVI) trial showed that newer generation self‐expandable and balloon‐expandable valves were equivalent as per the composite endpoint of all‐cause mortality, stroke, permanent pacemaker (PPM) implantation and paravalvular leakage.^40^ It should be noted that this trial only consisted of 447 patients,^40^ and thus larger studies such as the ongoing SMall Annuli Randomized to Evolut or SAPIEN Trial (SMART)^41^ are required to compare individual clinical endpoints and draw firmer conclusions.

The valve systems produced by Medtronic and Edwards Lifesciences are the most established in the TAVR market, but new competitors are emerging. Abbott previously gained Food and Drug Administration (FDA) approval for the self‐expanding Portico TAVR system,^42^ before developing the improved Navitor system that was approved by the FDA in 2023.^43^ Similarly, Boston Scientific initially developed the self‐expanding ACURATE neo device,^44^ but this was found to be inferior to the existing Sapien 3^45^ and CoreValve Evolut.^46^ systems. However, the subsequent ACURATE neo2 device demonstrated significant improvements^44^, ^47^ and received a CE mark in 2020, although it is still awaiting FDA approval.^48^ It remains to be seen whether these novel devices will become well‐established options for TAVR in the future.

## 3 PREPROCEDURAL EVALUATION OF PATIENT SUITABILITY FOR TAVR

A comprehensive assessment of patient suitability for valve replacement is conducted by a multidisciplinary heart valve team. Mechanical valves can only be implanted surgically and therefore a transcatheter approach is not appropriate for patients who are deemed more suitable for mechanical valve replacement.^49^ The surgical risk profile of a patient can be calculated using scoring systems, such as the Society of Thoracic Surgeons Predicted Risk of Mortality (STS‐PROM) score.^50^ or the European System for Cardiac Operative Risk Evaluation II (EuroSCORE II).^51^ Initial TAVR trials were conducted on patients who were inoperable or had a high surgical risk profile (often defined as an STS‐PROM/EuroSCORE II >8%–10%)^50^, ^51^ to explore the viability of TAVR as an alternative to surgery. Together with improvement in technology and center experience, its feasibility in lower and intermediate risk patient subgroups have since been explored and supported.^52^, ^53^

Age is important when evaluating the suitability of patients for TAVR; patients from older age groups may benefit from the procedure since it is less invasive compared to conventional open‐heart surgery.^13^, ^14^ Furthermore, a differential effect of patient sex on TAVR outcomes has been reported within the literature.^54^ Females have been reported to be more at risk of vascular complications compared to men; this is likely due to more tortuous iliofemoral vasculature and smaller luminal diameters.^54^, ^55^ Large‐scale observational studies have shown that female TAVR patients are often frailer and present with higher STS‐PROM risk scores at baseline.^54^ Although patient frailty is not included in the calculation of the STS‐PROM and EuroSCORE II scores, it serves as an independent predictor of mortality and complications during postprocedural recovery.^56^, ^57^, ^58^

The two main guidelines for choosing between SAVR and TAVR are the 2020 American College of Cardiology (ACC)/American Heart Association (AHA)^13^ and the 2021 European Society of Cardiology (ESC)/European Association for Cardio‐Thoracic Surgery (EACTS)^14^ guidelines. Both guidelines recommend SAVR for younger patients who are suitable for surgery whilst preferring TAVR for older inoperable patients, but the defined age ranges differ as shown by Table 1. There is a further subset of patients who are suitable for either TAVR or SAVR, and thus the assessment of individual characteristics and the balance between valve durability and life expectancy is crucial.^13^, ^14^

**Table 1:** American College of Cardiology (ACC)/American Heart Association (AHA), European Society of Cardiology (ESC)/European Association for Cardio‐Thoracic Surgery (EACTS).

2020 ACC/AHA guidelines	2021 ESC/EACTS guidelines
Transfemoral TAVR is preferred over SAVR for older patients (>80 years) or younger patients with a life expectancy <10 years.	TAVR is preferred over SAVR for older patients (>75 years), patients with a high surgical risk (STS‐PROM/EuroScore II >8%), or inoperable patients.
SAVR is preferred over TAVR for younger patients (<65 years) or patients with a life expectancy >20 years.	SAVR is preferred over TAVR for younger patients (<75 years) with a low surgical risk (STS‐PROM/EuroScore II <4%), or operable patients unsuitable for TAVR.

After deciding whether a patient is suitable for valve replacement and choosing between SAVR and TAVR, preprocedural assessment is also required to determine how the TAVR should be carried out and whether any concomitant procedures are necessary.

### 3.1 Sizing of the prosthetic

Unlike SAVR, where sizers could be used under direct visualization to determine the optimal fit of prosthesis, imaging is needed before TAVR to assess the aortic annulus diameter.^59^ An undersized prosthesis increases the risk of paravalvular regurgitation, which is seen in more than half of patients post‐TAVR.^60^ In contrary, an oversized prosthesis could impinge on nearby conductive tissue and predispose patients to arrhythmias.^61^ 3‐dimensional imaging modalities, such as multidetector computed tomography (MDCT), have over time superseded 2‐dimensional echocardiography to become the gold standard for assessing the aortic annulus size.^13^, ^14^ This has revolutionized the preprocedural workup for TAVR since 2‐dimensional echocardiography often underestimated the aortic annulus diameter, leading to prosthesis undersizing^62^, ^63^ and increased risks of paravalvular regurgitation. Yet, despite its clear advantages, MDCT requires administration of a contrast medium and therefore may not be suitable for patients with renal impairment or contrast allergies.^13^, ^14^ In such cases, 3‐dimensional TOE could be used as a viable alternative, with recent evidence showing that it correlates well with MDCT when assessing the aortic annulus parameters.^64^, ^65^

### 3.2 Valve and leaflet considerations

Assessment of valve calcification is an integral aspect of the preprocedural workup. Although some aortic valve calcification is beneficial for keeping the prosthetic valve in place, excessive calcification can impair prosthetic valve apposition and risk paravalvular regurgitation.^66^ Pushing the prosthetic through the diseased aortic valve can also displace any calcified deposits surrounding the leaflets; this can lead to embolisation and impair long term patient outcomes.^66^ Indeed, some authors have shown a correlation between the degree of calcification, based on the Agatston calcium score, and embolization risk.^66^, ^67^ In some scenarios, valve calcification can also lead to the fusion of native valve leaflets. This could hinder prosthetic valve expansion during valve deployment, which may further precipitate paravalvular regurgitation.^66^, ^67^

### 3.3 Coronary artery disease

Imaging enables the screening of coronary artery disease, which is commonly seen in conjunction with aortic stenosis.^68^ Some studies have reported its prevalence to exceed 60% in patients undergoing SAVR and around 50% in patients undergoing TAVR.^68^ Assessment of coronary artery disease plays an important role in the choice of management between surgery and TAVR. Open heart surgery may be preferred over TAVR if patients require additional cardiac surgeries, such as a coronary artery bypass graft, since surgery allows multiple procedures to be conducted in the same sitting.^69^ Although there is evidence for TAVR with concomitant coronary intervention as a potential alternative in patients who are suitable, more research is needed for firm conclusions to be made.^13^, ^14^, ^70^

Coronary artery disease is traditionally evaluated using invasive coronary angiography. Studies have consistently highlighted excellent negative predictive values for CT coronary angiography in ruling out significant CAD.^71^, ^72^, ^73^ Nonetheless, one drawback of CT coronary angiography is that its diagnostic performance could be hindered by coronary calcification, which is common in TAVR patients.^74^ CT coronary angiography has received considerable attention over time since it demands lower contrast volume, has good diagnostic accuracy and is less invasive compared to conventional angiography.^73^, ^75^ This has important clinical and cost implications because minimizing the invasiveness of preprocedural evaluation could shorten the duration of in‐hospital stay following TAVR.^73^

## 4 POSTPROCEDURAL IMAGING AND COMPLICATIONS

TTE remains as the modality of choice for postprocedural follow‐up, owing to its good diagnostic accuracy, ability to monitor haemodynamic status, absence of ionizing radiation and its extensive availability.^76^ Although minor discrepancies exist, guidelines in general recommend that patients should receive TTE at the following time points: (1) before hospital discharge, (2) 30 days post‐TAVR, (3) 1‐year post‐TAVR, and (4) yearly thereafter.^13^, ^77^, ^78^ TTE can be used to monitor postprocedural complications and assess the haemodynamic performance of the prosthetic.^14^ In addition, TTE is crucial in identifying cardiac structural changes following TAVR. Specifically, long‐term aortic stenosis commonly results in left ventricular hypertrophy; TAVR alleviates the left ventricular afterload and promotes left ventricular mass regression.^79^ It is therefore important to monitor these changes via echocardiographic assessment as they have been shown to be associated with patient rehospitalisation and survival rate following TAVR.^79^, ^80^

### 4.1 Vascular complications

The process of achieving vascular access for TAVR is a major cause of complications, such as pseudoaneurysm, haematoma, dissection, and perforation.^81^ As the procedure involves puncturing a hole in the artery to gain access, a closure device is required to seal the arteriotomy at the end of the procedure; failure of these closure devices will predispose patients to bleeding, which can be life‐threatening.^81^ A meta‐analysis, which defined outcomes in accordance with the Valve Academic Research Consortium criteria, found that TAVR with first‐generation valves resulted in a major vascular complication in approximately 12% of patients, as well as an approximately 16% rate of life‐threatening bleeding.^82^ Notably, vascular complications and major bleeding are independently associated with poorer outcomes including a higher incidence of death at 30 days and 1 year, alongside an increased likelihood of hospitalization at 1 year.^83^

Despite the high incidence of vascular complications in early studies, this appears to be decreasing over time, with recent trials reporting rates below 5%.^52^, ^53^ Along with increased operator experience, a combination of factors has led to this improvement. First, newer generation TAVR devices possess smaller sheath diameters and flexible delivery systems, which reduces the degree of vascular trauma experienced during the procedure.^34^ A 2020 meta‐analysis revealed that significantly lower rates of vascular complications were associated with newer generation devices (5.42 ± 4.75%) compared to first‐generation devices (11.52 ± 7.23%).^84^ The other key factor has been the use of MDCT to assess the suitability of the peripheral vasculature, as this imaging modality enables the identification of important vascular pathologies at the access site, such as heavy calcification, which may increase the risk of complications.^85^ MDCT is also used to measure the minimal luminal diameter, which allows for the sheath to femoral artery ratio (SFAR) to be calculated^85^; high SFAR is a major risk factor for vascular complications in TAVR.^86^

### 4.2 Bioprosthetic valve failure

TAVR is performed using bioprosthetic valves, and thus patients may experience complications as a result of structural valve deterioration and eventual bioprosthetic valve failure.^87^ Although patients with a high life expectancy are usually offered surgery instead of TAVR due to concerns about durability, a significant number of TAVR patients still experience these complications during their lifetime. For example, a meta‐analysis found that the pooled incidence of structural valve deterioration in TAVR patients at 1 year was 4.93%.^88^ Given that the indications for TAVR are being expanded further to low‐risk patients with a longer life expectancy,^13^, ^14^ bioprosthetic valve failure could soon become a much bigger issue.

The current concerns regarding TAVR durability may be resolved as the devices continue to be improved with each generation.^89^ Otherwise, failed bioprosthetic valves can be managed with redo‐SAVR,^90^ but the high surgical risk profile of many TAVR patients limits the feasibility of this option. Valve‐in‐valve TAVR, which involves implanting a second device inside the initial prosthesis, has shown potential as an alternative to redo‐SAVR, with meta‐analyses highlighting similar mortality rates with the two procedures.^91^, ^92^ Moreover, the valve‐in‐valve procedure may be associated with a lower incidence of major bleeding and stroke, thus increasing its suitability for patients with a high surgical risk.^92^

### 4.3 Paravalvular aortic regurgitation

Paravalvular aortic regurgitation is commonly seen in patients following TAVR but refinements in the procedure and prosthesis design have dramatically reduced its incidence.^84^ According to a meta‐analysis conducted by Winter et al.,^84^ its occurrence has decreased from around 12% in first‐generation devices to about 2% in more recent studies. Although most cases are mild in severity, a significant proportion of patients experience moderate to severe regurgitation which are associated with increased mortality rates.^93^, ^94^ During TAVR, the prosthetic valve is often superimposed onto its diseased counterpart; this means that the procedure frequently yields an insufficient seal that allows blood to “leak” around the bioprosthesis.^95^ Furthermore, patients who need TAVR often come from older age groups and they therefore frequently co‐present with calcified aortic valves.^96^ Valve calcification interferes with the expansion and apposition of the bioprosthetic when it is being deployed, ultimately predisposing patients to paravalvular regurgitation.^97^

As aforementioned, preprocedural assessment is vital for mitigating the risk of paravalvular regurgitation, and the addition of outer skirts to newer‐generation valve systems has been a useful innovation. Additional periprocedural interventions to reduce the degree of paravalvular leakage include balloon postdilation to expand the valve further and achieve a better seal, which has shown promise for balloon‐expandable and self‐expandable valves.^98^, ^99^ Paravalvular regurgitation may otherwise be due to suboptimal positioning of the valve and, in certain cases, this can be treated by implanting a second valve using the valve‐in‐valve method.^100^ An Italian registry analysis concluded that the valve‐in‐valve technique is an effective option for managing acute paravalvular leakage without necessitating surgery.^100^

### 4.4 Cerebrovascular events (CVEs)

CVEs following TAVR are not uncommon, and they pose a significant clinical challenge for management. Patients are most vulnerable to CVEs during and immediately following the procedure (<24 h)^101^; importantly, CVEs during the first 30 days of recovery is associated with greater 30‐day postprocedural mortality, with some studies reporting a greater than sixfold increase in risk.^102^, ^103^ A crude generalization is that acute (≤24 h) and subacute (<30 days) CVEs are more likely to be associated with the TAVR procedure itself, during which debris can be displaced from the valve or the blood vessels.^101^ Conversely, CVEs that occur >30 days following the procedure are often associated with long‐standing comorbidities, such as chronic atrial fibrillation and peripheral vascular disease.^101^ However, current data on periprocedural CVEs frequently omit the timing at which these events have occurred, restricting the scope for further analysis.

The need to prevent periprocedural CVEs has inspired the design of cerebral embolic protection (CEP) systems such as the Sentinel.^104^ and Emblok^105^ devices. These are both filters which permit blood flow from the aorta, whilst removing embolic debris to prevent CVEs. A 2020 in‐man pilot study on 20 participants concluded that the Emblok system seems to be safe, and that the procedure is achievable,^105^ although larger studies should be conducted. The Sentinel CEP system is more established, gaining FDA approval in 2017,^104^ but a 2022 randomized control trial found that the use of this device did not significantly reduce the likelihood of stroke.^106^

Antithrombotic therapy is another vital component of CVE prevention in the postprocedural period. The most recent guidelines recommend the use of low dose aspirin monotherapy or dual antiplatelet therapy with aspirin and clopidogrel for 3–6 months following TAVR.^13^ However, TAVR patients can also experience bleeding complications, and hence the patient profile should influence the antithrombotic regimen. The POPular TAVI trial has provided some valuable insights regarding when to use different antithrombotic strategies.^107^, ^108^ The study found that for patients with no indication for anticoagulation, aspirin monotherapy reduced the risk of bleeding compared to a combination of aspirin and clopidogrel, without increasing the incidence of ischaemic events.^107^ Meanwhile, patients who had an requirement for oral anticoagulation suffered fewer severe bleeding events when receiving oral anticoagulation alone instead of oral anticoagulation with clopidogrel, and again there was no significant increase in major ischaemic events.^108^

### 4.5 Conductive abnormalities

Arrhythmias often result from the direct injury of the cardiac conduction tissue during the TAVR procedure, with some patients needing postintervention PPM implantation.^109^ The implanted prosthesis requires slight oversizing to ensure adequate anchorage and minimize the risk of postprocedural paravalvular aortic regurgitation.^110^ Nonetheless, it is often difficult to gauge the optimal degree of oversizing needed and “overshooting” could consequently traumatize adjacent conductive tissue.^110^

Although prior studies investigating the intersex differences in post‐TAVR complications have been conflicting,^111^, ^112^ a recent meta‐analysis has shown that men are more at risk of needing PPM implantation.^61^ This finding by Ullah et al.^61^ could be partly attributed to the greater preexisting comorbidity burden that male TAVR patients often present with.^113^, ^114^ Male patients also tend to receive implants of larger sizes as they generally have larger aortic annulus diameters compared to women, placing men at increased risk of conductive complications.^113^, ^114^ Pre‐existing conductive abnormality (acquired before TAVR) is another significant predictor of PPM implantation.^115^, ^116^, ^117^ In an analysis of the Placement of Aortic Transcatheter Valve (PARTNER) 1 trial, patients who received PPM implantation after TAVR were nearly four times more likely to have had pre‐existing right bundle‐branch block and nearly twice as likely to have had pre‐existing left anterior fascicular block when compared to patients who did not require PPM implantation^118^; similar findings were also reported in subsequent studies.^61^

On the other hand, some risk factors for PPM implantation can be controlled, such as the choice of prosthesis.^61^ Self‐expanding valves are traditionally associated with a greater risk of conductive abnormalities when compared to its balloon‐expandable counterpart.^84^, ^119^, ^120^ The reported incidence of PPM implantation post‐TAVR has ranged between 5% and 10% for the Edwards SAPIEN balloon‐expandable valves and approximately 25% for the self‐expanding Medtronic CoreValve.^40^, ^121^ However, the more recent SAPIEN 3 valve has been reported by some to exhibit higher rates of PPM dependency when compared to its predecessors. Despite reducing the risk of paravalvular aortic regurgitation, the design addition of an outer sealing skirt (Figure 2) likely increases the radial force exerted on surrounding cardiac tissue and predisposes patients to atrioventricular conductive disturbances.^122^

Recently, several studies have investigated whether the risk of PPM implantation can be mitigated by modifying the conventional TAVR procedure. Firstly, Sammour et al.^123^ demonstrated a novel, systematic method for deploying balloon‐expandable valves, and their findings suggest that reducing the depth of valve implantation from ~3.2 to ~1.5 mm may overcome the issues seen with the SAPIEN 3 valve. This novel method decreased the rate of PPM implantation at 30 days from 13.1% to 5%, and the incidences of complete heart block and left bundle branch block were also significantly lower.^123^ The results of this study inspired the use of a cusp‐overlapping technique for self‐expandable TAVR to achieve a high implantation position, and again the rates of PPM implantation were consequently lower.^124^ It should be noted that high implantation may theoretically increase the risk of valve embolisation and aortic regurgitation,^125^ but overall the safety profile of the cusp‐overlapping technique appears to be similar to the conventional approach.^124^, ^126^

## 5 MAJOR TAVR TRIALS

Several pivotal trials have compared TAVR with the conventional SAVR to evaluate factors such as efficacy and safety. These trials can also be classified based on the surgical risk profile of the selected patients, using the mean STS‐PROM score.^127^ For example, the patient that was selected for the first TAVR was considered to be a “last‐resort” case^6^ and, for the first few years, TAVR was exclusively performed on patients who were unsuitable for surgery.^128^ Due to innovations in technology, increased data and operator experience, there has been a growing interest in assessing the efficacy of TAVR in patients with lower risk profiles. Therefore, the participants in the PARTNER 1A^35^, ^129^ and 1B trials^130^, ^131^ which began in 2007 had a higher risk profile than those assessed in the PARTNER 3 trial^52^, ^132^ several years later. Table 2 summarizes the landmark TAVR trials that have been published to date.

**Table 2:** Placement of Aortic Transcatheter Valve Trial (PARTNER), Surgical Replacement and Transcatheter Aortic Valve Implantation (SURTAVI), Nordic Aortic Valve Intervention (NOTION).

Trial	Participant surgical risk	Comparison	Enrollment period	Valve type	Main findings
**PARTNER 1B**	Extreme risk and inoperable	TAVR vs. standard therapy	2007–2009	Edwards SAPIEN *Balloon‐expandable*	TAVR was associated with significantly reduced all‐cause mortality after 1 and 5 years compared to standard medical therapy. However, TAVR was associated with increased major vascular complications and major bleeding.
**CoreValve ER**	Extreme risk and inoperable	TAVR only	2011–2012	Medtronic CoreValve *Self‐expandable*	The rate of all‐cause death or major stroke following TAVR was 26.0% after 1 year and 38.0% after 2 years. Negative outcomes of participants were mainly due to comorbidities rather than valve issues.
**PARTNER 1A**	High risk	TAVR vs. SAVR	2007–2009	Edwards SAPIEN *Balloon‐expandable*	TAVR was noninferior to SAVR in terms of all‐cause mortality after 1 and 5 years. However, TAVR was associated with increased major vascular complications.
**CoreValve HR**	High risk	TAVR vs. SAVR	2011–2012	Medtronic CoreValve *Self‐expandable*	TAVR was associated with significantly reduced all‐cause mortality after 1 and 2 years compared to SAVR. However, TAVR was associated with increased major vascular complications and permanent pacemaker implantations after 1 year.
**PARTNER 2**	Intermediate risk	TAVR vs. SAVR	2011–2013	Edwards SAPIEN XT *Balloon‐expandable*	TAVR was noninferior to SAVR in terms of all‐cause mortality or disabling stroke after 2 and 5 years. However, TAVR was associated with increased major vascular complications.
**SURTAVI**	Intermediate risk	TAVR vs. SAVR	2012–2016	Medtronic CoreValve, Medtronic Evolut R *Self‐expandable*	TAVR was noninferior to SAVR in terms of all‐cause mortality or disabling stroke after 2 years. However, TAVR was associated with increased rehospitalisation and aortic valve reintervention after 2 years.
**NOTION**	Low risk	TAVR vs. SAVR	2009–2013	Medtronic CoreValve *Self‐expandable*	TAVR was noninferior to SAVR in terms of all‐cause mortality, stroke, and myocardial infarction after 1, 5 and 10 years. However, TAVR was associated with increased aortic regurgitation after 1 year, as well as higher rates of paravalvular regurgitation and aortic reintervention after 5 years. Nevertheless, TAVR appears to demonstrate statistically better durability after 10 years compared to SAVR.
**PARTNER 3**	Low risk	TAVR vs. SAVR	2016–2017	Edwards SAPIEN 3 *Balloon‐expandable*	TAVR was associated with significantly reduced all‐cause mortality, stroke, or rehospitalisation after 1 and 2 years compared to SAVR. After 5 years, the incidence of this composite endpoint was similar in the two groups. However, TAVR was associated with a higher rate of mild paravalvular regurgitation and new left bundle‐branch block.
**Evolut LR**	Low risk	TAVR vs. SAVR	2016–2018	Medtronic CoreValve, Medtronic Evolut R, Medtronic Evolut PRO *Self‐expandable*	TAVR was noninferior to SAVR in terms of all‐cause mortality or disabling stroke after 2, 3, and 4 years. However, TAVR was associated with increased permanent pacemaker implantations.

*Note*: Extreme risk = inoperable. High risk = STS‐PROM ≥8%. Intermediate risk = STS‐PROM ≥4%. Low risk = STS‐PROM <4%.

The trials which fall under the extreme‐risk category consist of the PARTNER 1B^130^, ^131^ and CoreValve Extreme Risk (CoreValve ER)^133^ trials. The participants of these trials were deemed inoperable, so TAVR was compared with standard medical therapy or an objective performance goal. The PARTNER 1B trial found that TAVR performed with the first‐generation Edwards Sapien heart‐valve system superseded standard medical therapy with an absolute risk reduction in all‐cause mortality of over 20% after 1^130^ and 5 years,^131^ despite the increased incidence of neurovascular events at 30 days and 1 year.^130^ This positive finding was supported by the single‐arm nonrandomised CoreValve ER trial which investigated the Medtronic self‐expanding CoreValve prosthesis instead.^133^ In this study, the rate of all‐cause mortality or major stroke following TAVR was 26.0% after 1 year and 38.0% after 2 years, but these negative outcomes were primarily due to the participants’ co‐morbidities rather than valve performance.^133^

The PARTNER 1A^35^, ^129^ and CoreValve U.S. Pivotal High Risk (CoreValve HR)^36^ trials assessed the efficacy of TAVR in patients from the high‐risk category (STS‐PROM ≥8%). In these trials, SAVR was considered a viable alternative and, therefore, it was possible to compare TAVR with SAVR. A total of 699 patients were enrolled into the PARTNER 1A trial which assessed the balloon expandable Edwards Sapien valve, and the results showed TAVR to be noninferior to SAVR in terms of all‐cause mortality at 30 days, 1^35^ and 5 years.^129^ However, TAVR was associated with a greater incidence of CVEs than SAVR after 30 days and 1 year.^35^ Although the differences in risk diminished after 5 years,^129^ these early findings highlighted the potential room for technological (e.g., embolic protection devices) and procedural adaptations to improve patient outcomes in the years to come. Following PARTNER 1A, the randomized CoreValve HR trial found that the rate of 1‐ and 2‐year all‐cause mortality was significantly lower in the TAVR group than the SAVR group, making it the first trial to show the potential superiority of TAVR over conventional surgery.^36^

As TAVR became more widely approved, there was a growing desire to expand its indications to lower risk groups. Consequently, the risk profile of trial participants started to decrease. The most notable trials in the intermediate‐risk category (STS‐PROM ≥4%) are the PARTNER 2^134^ and Surgical Replacement and Transcatheter Aortic Valve Implantation (SURTAVI)^135^ trials, with mean STS‐PROM scores of 5.8% and 4.5%, respectively. The primary finding from the PARTNER 2 trial (Edwards Sapien XT valve) was that the rate of all‐cause mortality or disabling stroke at 2 years from TAVR and SAVR was similar, although there was a lower incidence of major vascular complications and paravalvular aortic regurgitation in the surgical patients.^134^ Further analysis of the trial published in 2020 concluded that the 5‐year outcomes of both procedures were also similar in regard to mortality and disabling stroke; however, re‐hospitalizations and aortic valve re‐interventions were more common after TAVR than SAVR.^136^ Meanwhile, the SURTAVI trial supported these findings, albeit using the CoreValve and Evolut R bioprostheses instead.^135^

Last, the use of TAVR in patients with a low surgical risk profile (STS‐PROM <4%) was evaluated by the PARTNER 3,^52^, ^132^ Evolut Low Risk (Evolut LR)^53^, ^137^ and Nordic Aortic Valve Intervention (NOTION)^138^, ^139^, ^140^ trials. The PARTNER 3 trial^52^, ^132^ randomly assigned 1000 patients to either TAVR, performed using the Edwards Sapien 3 system, or SAVR. Analysis showed that the rate of the composite of death, stroke, or rehospitalisation after 1^52^ and 2 years^132^ was significantly lower in the TAVR group than with SAVR. After 5 years, the incidence of this composite endpoint was similar in the two groups.^141^ Contrary to previous studies,^35^, ^134^ TAVR was not associated with an increased incidence of moderate or severe paravalvular regurgitation in the PARTNER 3 trial.^52^, ^132^ Additionally, the TAVR patients had shorter index hospitalizations suggesting that the procedure could be cost‐effective.^52^ The Evolut LR trial also found that TAVR with either the CoreValve, Evolut R or Evolut PRO was noninferior to SAVR based on the composite endpoint of death or disabling stroke after 2,^53^ 3,^137^ and 4 years,^142^ with the difference between the two groups increasing over time in favor of TAVR.^143^ The longest follow‐up data comparing TAVR and SAVR in low surgical risk patients was collected in the NOTION trial, which reported no statistically significant differences in all‐cause mortality, stroke and myocardial infarction at 5^138^ and 10 years.^140^ Moreover, TAVR appears to demonstrate lower rates of moderate or greater structural valve deterioration and bioprosthetic valve dysfunction after 10 years compared to SAVR, according to data presented in 2023.^140^

## 6 FUTURE DIRECTIONS

Over the best part of two decades, the TAVR procedure has evolved tremendously and is becoming a safe and viable alternative to surgery. The popularity of TAVR is likely to accrue over time because of increasing data and new technological innovations. Examples of this include the aforementioned development of CEP devices to address the challenge of peri‐procedural CVEs.^104^, ^105^ Furthermore, in response to the higher reported incidence of paravalvular regurgitation following TAVR than SAVR,^36^, ^129^, ^134^ solutions are being worked on such as novel “occluders” which are placed around the prosthetic valve to prevent leakage, and these have shown potential in vitro.^144^

TAVR may also become more popular because of expanding indications. Although an initial study evaluating the use of TAVR for pure native aortic regurgitation found that many patients required second valve implantation or experienced residual regurgitation,^145^ newer generation devices have shown more positive results.^146^ Furthermore, aortic valve replacement has typically been reserved for patients with severe aortic stenosis,^13^, ^14^ but recent observational data has suggested that moderate aortic stenosis is enough to significantly worsen patient mortality, especially when left ventricular ejection fraction is reduced.^147^, ^148^ These findings have sparked interest into a potential role for TAVR in the management of nonsevere aortic stenosis, with retrospective studies showing promising results.^148^, ^149^ This topic is being further explored by the ongoing TAVR UNLOAD,^150^ Evolut EXPAND TAVR II Pivotal^151^ and PROGRESS^152^ randomized trials.

Valve durability is still a concern regarding the use of TAVR in low‐risk patients. A study estimated that in low‐risk patients with a mean age of 73, TAVR valves need to be at least 30% as durable as surgical valves to prevent a reduction in life expectancy; however, this threshold is higher for younger patients.^153^ Future long‐term data on valve durability is required to improve confidence over the use of TAVR in low‐risk patients from younger age groups. Another key challenge is identifying the optimal post‐TAVR anticoagulation regimen.^154^ Oral anticoagulants are currently the standard therapy,^154^ but trials are being conducted to compare direct oral anticoagulants with vitamin K antagonists.^155^, ^156^ Establishing clarity over this aspect of postprocedural care will be an important future objective.

The growing success of TAVR naturally raises the question: can transcatheter techniques be used to replace other valves? The mitral valve is oval‐shaped and saddle‐like so developing a transcatheter mitral valve replacement (TMVR) has been difficult. A meta‐analysis conducted by Takagi et al.^157^ found that TMVR was associated with elevated mortality compared to predicted operative mortality, but a key limitation was that the included studies lacked the control of conventional mitral valve surgery. In January 2020, Abbott's Tendyne TMVR device became the first to receive a CE mark and subsequent approval for use in Europe.^158^ Transcatheter approaches for the replacement of tricuspid^159^, ^160^ and pulmonary^161^ valves are being developed and studied as well, but they are both significantly behind TAVR in terms of progress to date.

## 7 CONCLUSION

Since its inception in 2002, the TAVR procedure has made striking advances. Minimally invasive procedures have become the dominant forms of treatment for a variety of conditions. Challenges relating to durability, complications and cost still need to be overcome, but TAVR will continue to generate excitement in the field of interventional cardiology for years to come.

## CONFLICT OF INTEREST STATEMENT

The authors declare no conflicts of interest.

## Data Availability

Data sharing not applicable to this article as no datasets were generated or analysed during the current study.
